# Keep an Ear Out for *Francisella tularensis*: Otomastoiditis Cases after Canyoneering

**DOI:** 10.3389/fmed.2016.00009

**Published:** 2016-03-03

**Authors:** Brice Guerpillon, Andre Boibieux, Clemence Guenne, Christine Ploton, Tristan Ferry, Max Maurin, Emmanuel Forestier, Olivier Dauwalder, Patrick Manipoud, Aicha Ltaïef-Boudrigua, Robert Gürkov, Francois Vandenesch, Coralie Bouchiat

**Affiliations:** ^1^Infectious Diseases Department, Hospices civils de Lyon, Lyon, France; ^2^ENT Department, Hospices civils de Lyon, Lyon, France; ^3^Bacteriology Laboratory, Hospices civils de Lyon, Lyon, France; ^4^French National Reference Center for Francisella tularensis, Grenoble, France; ^5^Infectious Diseases Department, Centre Hospitalier Métropole Savoie, Chambéry, France; ^6^ENT Department, Centre Hospitalier Métropole Savoie, Chambéry, France; ^7^Radiology Department, Hospices civils de Lyon, Lyon, France; ^8^ENT Department, Ludwig-Maximilians-Universität München, Munich, Germany

**Keywords:** *Francisella tularensis*, otitis media, otomastoiditis, canyoneering, France

## Abstract

We report here three unusual cases of otomastoiditis due to *Francisella tularensis*, complicated by cervical abscesses and persistent hearing loss, plus facial paralysis for one patient. Intriguingly, the three patients had practiced canyoneering independently in the same French river, between 2009 and 2014, several days before clinical symptoms onset. The results point out that fresh water exposure may be a potential contamination route for tularemia. Besides, due to the frequent complications and sequelae, we believe that *F. tularensis* should be considered as a possible etiology in case of otitis media, failure of the conventional antibiotic treatment, and suspicious exposure of the bacteria.

## Introduction

We here report the first series of culture and/or PCR – documented *Francisella tularensis* otomastoiditis. Intriguingly, the three patients had all independently practiced canyoneering between 2008 and 2014 in the same French river several days before clinical signs onset.

### Patient 1

The observation has already been published as a single case report by Gürkov et al. (1), but the epidemiologic link with the present series was uncovered by Guerpillon and Gürkov when retrospectively questioning the patient. Briefly, in summer 2008, a 27-year-old female with no comorbidity presented with acute otitis media and retropharyngeal swelling after 5 weeks of empirical treatment by subsequent amoxicillin, cefuroxim, and ceftriaxone. Computed Tomography scan (CT-scan) revealed a retropharyngeal abscess and mastoiditis.

### Patient 2

The following summer (2009), a 39-year-old female with no known comorbidity presented to an Ear Nose and Throat (ENT) emergency unit for vestibular disorders, conductive deafness, and fever. She reported being under clavulanic acid–amoxicilin for the past 2 weeks due to a sore throat with adenopathies. CT-scan confirmed the unilateral right otitis media and mastoiditis.

### Patient 3

**Figure 1:**
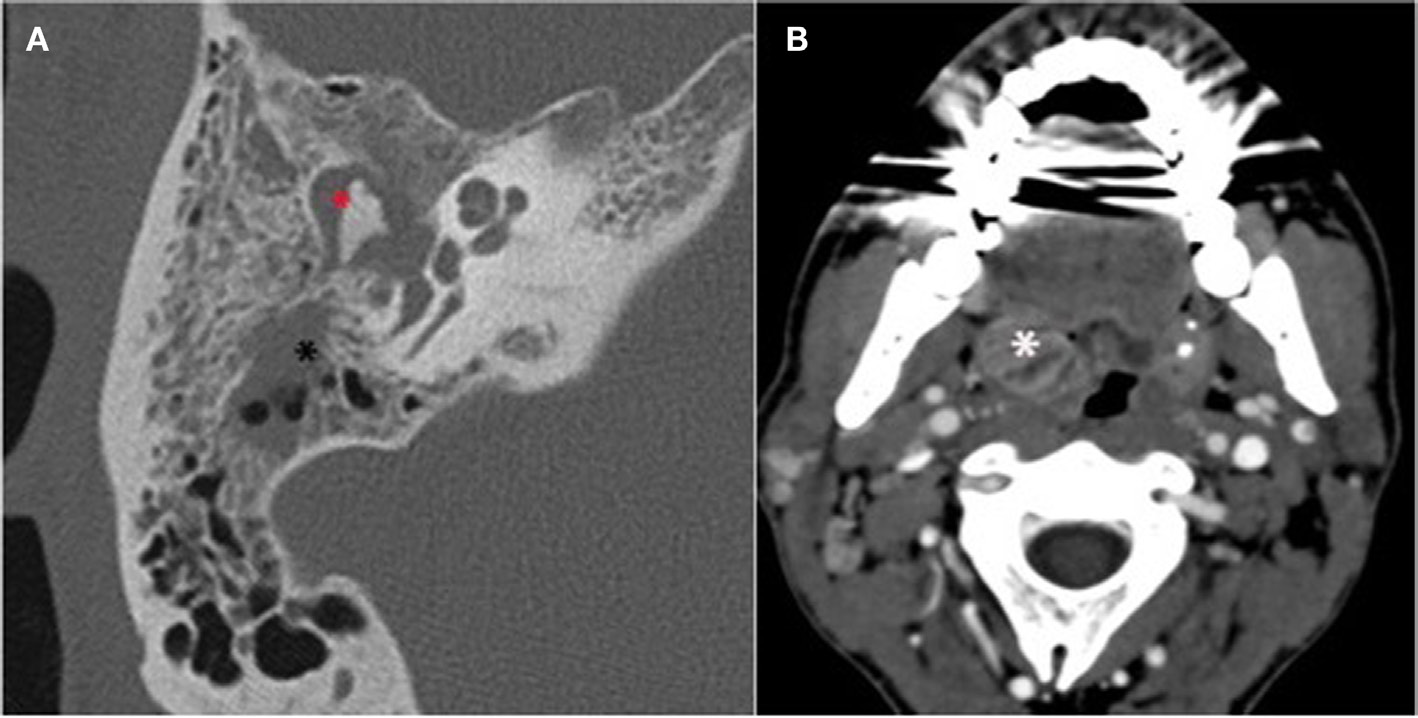
**Mastoid CT scan of patient 3**. **(A)** High-resolution axial section focus of the right temporal bone. Acute otitis media with tympanomastoid homogenous opacity inflammatory filling of the whole tympanic cavity (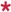) and mastoid cells (*). **(B)** Swelling related to the right tonsillitis (*).

The third case occurred in summer 2014 and involved a 43-year-old canyoneering instructor with no notable medical history. After 5 days suffering from right otalgia with accompanying hearing loss and asthenia, the patient received an empirical course of oral amoxicillin followed by oral cefpodoxim and ibuprofen from his general practitioner. No clinical improvement was observed. Thirteen days after the onset of clinical signs, the patient began suffering from right facial paralysis and thus consulted to an ENT department. Upon clinical examination, homolateral sensorineural deafness and right-side tonsilitis with a subdigastric adenopathy were noted. Peripheral grade 6 facial paralysis was confirmed (2). Otoscopy of the right side showed inflammatory stenotic external auditory canal. A paracentesis was performed, leading to a purulent discharge. Biological examination revealed an inflammatory syndrome (Table 1). CT-scan (Figure 1) confirmed the acute otitis media of the right ear, with blockage of the middle ear and the tympanic cavity, partial blockage of the mastoid cells, and the internal portion of the external auditory canal, as well as a lateral pharyngeal adenopathy on the right side (16 mm × 19 mm) and tonsillitis. Upon hospital admission, piperacillin–tazobactam (12 g/day) with a daily injection of amikacin (15 mg/kg) and topical ofloxacin (200 mg/day) treatment was initiated. Prednisone (1 mg/kg/day, 6 days) was added to treat the facial paralysis.

**Table 1:** **Characteristics of patients in a series of *Francisella tularensis* subsp. *holarctica* otomastoiditis**.

	Patient 1 (1)	Patient 2	Patient 3
Date	July 2008	July 2009	May 2014
Gender	Female	Female	Male
Age (years old)	27	39	43
Incubation (days)	<7	10	3–7
Clinical presentation	Pharyngitis	Pharyngitis	Tonsillitis (right-side)
	Purulent left otitis media	Purulent right otitis media	Purulent right otitis media
	Retropharyngeal swelling	Submandibular adenopathy	Subdigastric adenopathy
Complications	Cervical phlegmonous inflammation Conductive deafness (15–30 dB) Retropharyngeal abscess Mastoiditis	Conductive deafness (40 dB) Mastoiditis Vestibular disorders	Facial peripheral paralysis Mixt deafness (40 dB in low pitch and 80 dB in high pitch) Mastoiditis
CRP upon hospital admission	36 mg/L	90 mg/L	155 mg/L
Empirical treatment active for *Francisella tularensis*	None	Ofloxacin (8 days)	Amikacin (5 days)
Elapsed time between clinical signs onset and diagnosis (days)	44	25	28
Microbiological diagnostic techniques	16S PCR Serology	Culture	Culture
		*Francisella tularensis-*specific PCR	16S PCR
			*Francisella tularensis*-specific PCR
Bacterial etiology	*Francisella tularensis* subsp. *holarctica*	*Francisella tularensis* subsp. *holarctica*	*Francisella tularensis* subsp. *holarctica*
Treatment (from diagnosis)	Day 1–5: doxycyclin	Day 0–4: gentamicin	Day 0–45: ciprofloxacin + doxycycline
	Day 1–23: ciprofloxacin	Day 0–25: ciprofloxacin	Day 45–78: no treatment
	Day 11–23: gentamicin	Day 89: transtympanic tube	Day 78–108: doxycyclin
	Paracentesis				
	Lesions needle aspiration		
	Surgical drainage		
Outcome	Conductive deafness (8-month follow-up)	Conductive deafness (10 dB) minor vestibular disorders (10-month follow-up)	Asymptomatic (2-month follow-up)

## Background

*Francisella tularensis* is a bacterial agent of pleomorphic anthropozoonoses, such as ulceroglandular or oropharyngeal syndromes and pneumonia, depending on the route of inoculation. Acute otitis tularemia is excessively rare; only two cases have been described so far (1, 3). Contaminated water is known as the route of oropharyngeal infection when ingested (4–7). However, to our knowledge, direct skin contact when bathing has never been pointed out as the source of infection.

## Discussion

### Patient 1

16S rDNA polymerase chain reaction (16S PCR) testing of an adenopathy aspirated fluid revealed the presence of *F. tularensis* subsp *holarctica* DNA. The positive serology performed concomitantly corroborated the results, leading to a final diagnosis 44 days after the onset of the symptoms. Adapted antibiotic treatment (Table 1) combined with paracentesis and repeated needle aspirations of the suppurative lesions remained inefficient. Only open surgical drainage allowed to cure the infection. Finally, the patient, a music teacher, suffered from a lasting conductive deafness of 15–30 dB (1).

The patient reported canyoneering practice in the river Le Grenant during her journey to France, 7 days before the clinical onset.

### Patient 2

Bacteriological analysis of an otorrhea sample yielded monomicrobial culture of *F. tularensis* after 15 days incubation (i.e., 25 days after the onset of symptoms). The expertise of the French National Reference Center for *Francisella* allowed the identification of the subspecies *holarctica* by specific PCR. A first 1-week course of oral ofloxacin (200 mg twice a day) leading to partial clinical improvement only, a combined treatment with oral ciprofloxacin for 25 days and gentamicin for 5 days was initiated (Table 1). Ten months after the end of treatment, conductive deafness (10 dB) and minor vestibular disorders still remained.

Canyoneering in the river Le Grenant 10 days before the onset of the symptoms was the only exposure factor revealed by the medical history.

### Patient 3

The bacteriological analysis of the paracentesis pus sample yielded a monomicrobial culture of Gram negative bacilli after 3 days incubation, identified as *F. tularensis* by 16S PCR (4), and confirmed as *F. tularensis* subsp. *holartica* by the French National Reference Center using specific PCR. The facial paralysis, angina, and asthenia regressed after 5 days of amikacin/piperacillin–tazobactam treatment; the subdigastric adenopathy diminished while persistent. Antibiotic treatment was then adapted with oral ciprofloxacin and doxycycline plus topical ofloxacin for 45 days. Regarding the hearing loss, repeated audiometric reports revealed improvement of conductive audition despite no restitution *ad integrum*. After 45 days, the patient could not stand anymore the lack of physical activity imposed by fluoroquinolone administration and ceased his treatment. Due to the diagnosis delay and the initial loco-regional extension, he finally complied with the medical team suggestion for another 30 days of doxycycline.

**Figure 2:**
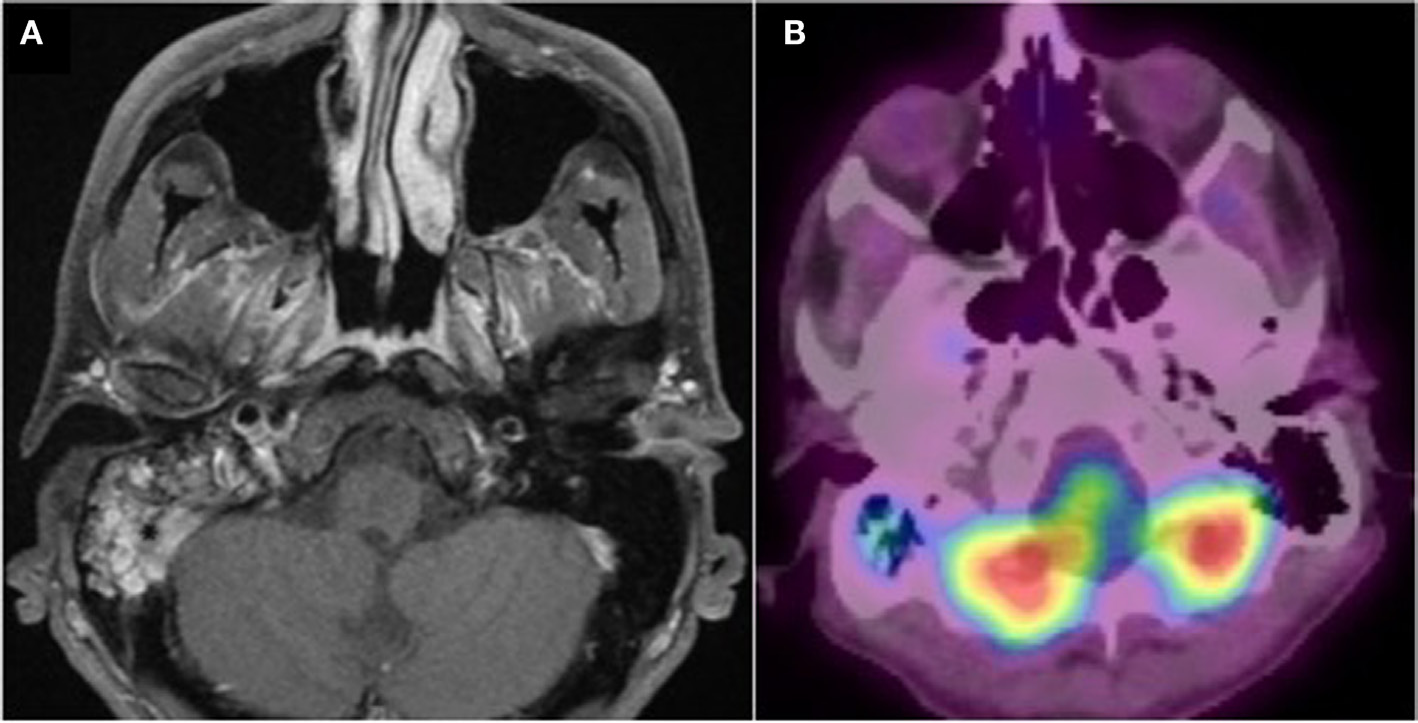
**Post treatment imaging**. **(A)** Gadolinium-enhanced axial T1 weighted fat-suppressed MRI. Minimal persistence of gadolinium enhancement in the right mastoid. **(B)** PET CT fused image. Good aeration and minimal persistence of 18 FDG uptake in the right mastoid. No other associated bone or tissue anomaly.

The follow-up MRI (Figure 2) and PET scan after 3 months showed satisfactory evolution with a minimal persistence of gadolinium enhancement in the right mastoid, and moderate hyper fixation of the right mastoid cells, respectively, with no other associated bone or tissue anomaly. The patient felt perfectly healthy. Noticeably, as a canyoneering instructor, the patient reported sessions in Le Grenant river (France) 3–7 days before the first clinical signs.

## Concluding Remarks

We report here the first series of three cases of *F. tularensis* otomastoiditis, which is an extremely rare localization, and may be related to an aquatic inoculation. Noticeably, the clinical presentation included severe deafness for all patients, possibly with retropharyngeal abscess and/or facial paralysis. Besides, the three cases noticeably occurred independently between 2008 and 2014 after a common exposure to the same French river (Le Grenant) 3–10 days before the clinical symptoms onset, which is consistent with the mean incubation period (3–5 days) (8). Given the very low incidence of tularemia in France, it is thus likely that the river was the common source of infection to the three cases.

Transmission occurs by ingestion, inhalation, or contact between contaminated liquids and human mucus. Moreover, *F. tularensis* is hosted in rodents and lagomorphs, and can be introduced into watercourses from animal carcasses (9, 10). Strikingly, a tannery was found to be closely located upstream from the canyoneering spot on the river border. Following the report of the case 2 (while not knowing the link of case 1 with the same river), the health authorities initiated a field survey and discovered that wastewater was discharged to the river. Bacteriological analyses of sewage and river water did not detect the presence of *Francisella* DNA, although only small volumes of water samples could be analyzed leading to a possible lack of sensitivity.

Given the complications observed due to the delay of diagnosis and the ineffectiveness of beta-lactams, we insist on this possible etiology in case of otitis media associated with fresh-water ­exposure and persisting after conventional antibiotic courses.

## Author Contributions

BG, AB, and TF are the infectious diseases specialists who examined patient 3 and gave advice for the antibiotic treatment and follow up. BG called the practitioner of case 1 (RG) and uncovered the epidemiological link with patient 3. He also wrote the article in collaboration with CB and FV. CG is the ENT specialist in charge of case 3. AL-B is the radiologist who diagnosed the otomastoiditis of case 3 and supplied the figures in the article. CP, CB, and OD are the microbiologists who performed the PCR analysis for case 3, allowing the diagnosis of tularemia. MM, as the director of the National Reference Centre for tularemia, gave his expertise for the confirmation of the three cases. He was also involved in the field survey. EF and PM are the infectious diseases and ENT specialists, respectively, who were in charge of case 2, and who alerted the National Reference Centre for tularemia. RG is the ENT specialist who diagnosed case 1 and collaborated with BG to retrospectively investigate case 1 journey in France, revealing the canyoneering practice in the same river. FV and CB initiated the project, gave overall guidance in the global study and wrote the article. All the authors actively contributed to the data compiling, bibliography search, read, and proposed corrections to the manuscript.

## Conflict of Interest Statement

The authors declare that the research was conducted in the absence of any commercial or financial relationships that could be construed as a potential conflict of interest.

## References

[1] Gürkov R Kisser U Splettstösser W Hogardt M Krause E. Tularaemia of middle ear with suppurative lymphadenopathy and retropharyngeal abscess. J Laryngol Otol (2009) 123:1252–7. 10.1017/S0022215109004757 19250590

[2] House JW Brackmann DE Facial nerve grading system. Otolaryngol Head Neck Surg (1985) 93:146–7. 3921901 10.1177/019459988509300202

[3] Luotonen L Tapiainen T Kallioinen M Luotonen J. Tularemia of the middle ear. Pediatr Infect Dis J (2002) 21:264–5. 10.1097/00006454-200203000-00024 12005098

[4] Maurin M Gyuranecz M Tularaemia: clinical aspects in Europe. Lancet Infect Dis (2016) 16:113–24. 10.1016/S1473-3099(15)00355-2 26738841

[5] Larssen KW Afset JE Heier BT Krogh T Handeland K Vikøren T Outbreak of tularaemia in central Norway, January to March 2011. Euro Surveill (2011) 16:19828. 21489376

[6] Reintjes R Dedushaj I Gjini A Jorgensen TR Cotter B Lleftucht A Tularemia outbreak investigation in Kosovo: case control and environmental studies. Emerg Infect Dis (2002) 8:69–73. 10.3201/eid0801.010131 11749751 PMC2730257

[7] Willke A Meric M Grunow R Sayan M Finke EJ Splettstösser W An outbreak of oropharyngeal tularaemia linked to natural spring water. J Med Microbiol (2009) 58:112–6. 10.1099/jmm.0.002279-0 19074661

[8] Dennis DT Inglesby TV Henderson DA Bartlett JG Ascher MS Eitzen E Tularemia as a biological weapon: medical and public health management. JAMA (2001) 285(21):2763–73. 10.1001/jama.285.21.2763 11386933

[9] Carvalho CL Lopes de Carvalho I Zé-Zé L Núncio MS Duarte EL. Tularaemia: a challenging zoonosis. Comp Immunol Microbiol Infect Dis (2014) 37:85–96. 10.1016/j.cimid.2014.01.002 24480622 PMC7124367

[10] Petersen JM Mead PS Schriefer ME. *Francisella tularensis*: an arthropod-borne pathogen. Vet Res (2009) 40:7. 10.1051/vetres:2008045 18950590 PMC2695023

